# Short- and long-term humoral immune response against *Yersinia pestis* in plague patients, Madagascar

**DOI:** 10.1186/s12879-020-05565-8

**Published:** 2020-11-10

**Authors:** Voahangy Andrianaivoarimanana, Alice Lantoniaina Iharisoa, Lila Rahalison, Marie Laurette Ralimanantsoa, Maherisoa Ratsitorahina, Rado J. L. Rakotonanahary, Elisabeth Carniel, Christian Demeure, Minoarisoa Rajerison

**Affiliations:** 1 Plague Unit, Institut Pasteur de Madagascar, 101 Antananarivo, Madagascar; 2 Central Laboratory for Plague, Ministry of Public Health, 101 Antananarivo, Madagascar; 3 Epidemiology Unit, Institut Pasteur de Madagascar, 101 Antananarivo, Madagascar; 4 Yersinia Research Unit, Institut Pasteur, 75724 Paris, France

**Keywords:** *Yersinia pestis*, Immune response, Plague, Human, Madagascar

## Abstract

**Background:**

Plague, a fatal disease caused by the bacillus, *Yersinia pestis*, still affects resources-limited countries. Information on antibody response to plague infection in human is scarce. Anti-F1 Ig G are among the known protective antibodies against *Y. pestis* infection. As a vaccine preventable disease, knowledge on antibody response is valuable for the development of an effective vaccine to reduce infection rate among exposed population in plague-endemic regions. In this study, we aim to describe short and long-term humoral immune responses against *Y. pestis* in plague-confirmed patients from Madagascar, the most affected country in the world.

**Methods:**

Bubonic (BP) and pneumonic plague (PP) patients were recruited from plague- endemic foci in the central highlands of Madagascar between 2005 and 2017. For short-term follow-up, 6 suspected patients were enrolled and prospectively investigated for kinetics of the anti-F1 IgG response, whereas the persistence of antibodies was retrospectively studied in 71 confirmed convalescent patients, using an ELISA which was validated for the detection of plague in human blood samples in Madagascar.

**Results:**

Similarly to previous findings, anti-F1 IgG rose quickly during the first week after disease onset and increased up to day 30. In the long-term study, 56% of confirmed cases remained seropositive, amongst which 60 and 40% could be considered as high- and low-antibody responders, respectively. Antibodies persisted for several years and up to 14.8 years for one individual. Antibody titers decreased over time but there was no correlation between titer and time elapsed between the disease onset and serum sampling. In addition, the seroprevalence rate was not significantly different between gender (*P* = 0.65) nor age (*P* = 0.096).

**Conclusion:**

Our study highlighted that the circulating antibody response to F1 antigen, which is specific to *Y. pestis*, may be attributable to individual immune responsiveness. The finding that a circulating anti-F1 antibody titer could persist for more than a decade in both BP and PP recovered patients, suggests its probable involvement in patients’ protection. However, complementary studies including analyses of the cellular immune response to *Y. pestis* are required for the better understanding of long-lasting protection and development of a potential vaccine against plague.

## Background

Plague is caused by the bacterium *Yersinia pestis*, and is a zoonotic disease mainly affecting rodents. Humans are occasionally infected through fleabites, thereby causing bubonic plague (BP) or by inhalation of infectious droplets leading to pneumonic plague (PP); the rarest but most contagious form. Plague was introduced to Madagascar in 1898 via steam ships at the east port of Toamasina. It reached the central highlands in 1921 and since then remained endemic in two traditional foci located in the central and northern highlands above 800 m of elevation [1]. Plague re-emerged in the west port city of Mahajanga in 1991. Presently, plague continues to persist in 5 countries in the world and Madagascar is the worst affected [2]. A large outbreak of PP in Madagascar in 2017 [3] attracted extensive attention and raised the possibility of an outbreak evolution in an endemic region. Given the significant public health risk of re-emergence of PP in an urban area and its potential for rapid spread, and the threat of weaponization of *Y. pestis,* there is an urgent need for a vaccine to provide enduring protection and for clinically proven effective antibiotic therapy. Further, the emergence of antibiotic-resistant *Y. pestis* strains has previously been documented in Madagascar [4] and to date there is no readily available licensed vaccine for plague. *Y. pestis* expresses a specific capsule-like surface antigen, the fraction 1 protein or F1 antigen which is synthesized in vivo in large quantities at 37 °C [5]. F1 antigen is highly immunogenic and was reported to confer anti phagocytic properties [6]. Anti-F1 antibodies have been widely used for serological diagnosis of plague infection [7–9]. They are known to be among the protective antibodies against *Y. pestis* infection [10]. Reports on short and long-term persistence of antibodies against *Y. pestis* among plague recovered patients are scarce [9, 11]. Knowledge of the humoral immune response from confirmed plague patients would be valuable to improve the development of an effective vaccine. Also, understanding the antibody kinetics has become of increasing importance for the potential use of serology as diagnostic tool. Indeed, due to various constraints, the confirmation rate during the last 2017 PP outbreak was very low [3], serology would have helped to confirm more cases.

In this study, we aimed (1) to follow the kinetics of antibodies against *Y. pestis* over a period of 1 month and (2) to determine the persistence of these antibodies in convalescent plague patients in Madagascar up to 180 months after infection.

## Methods

### Study design and setting

**Fig. 1:**
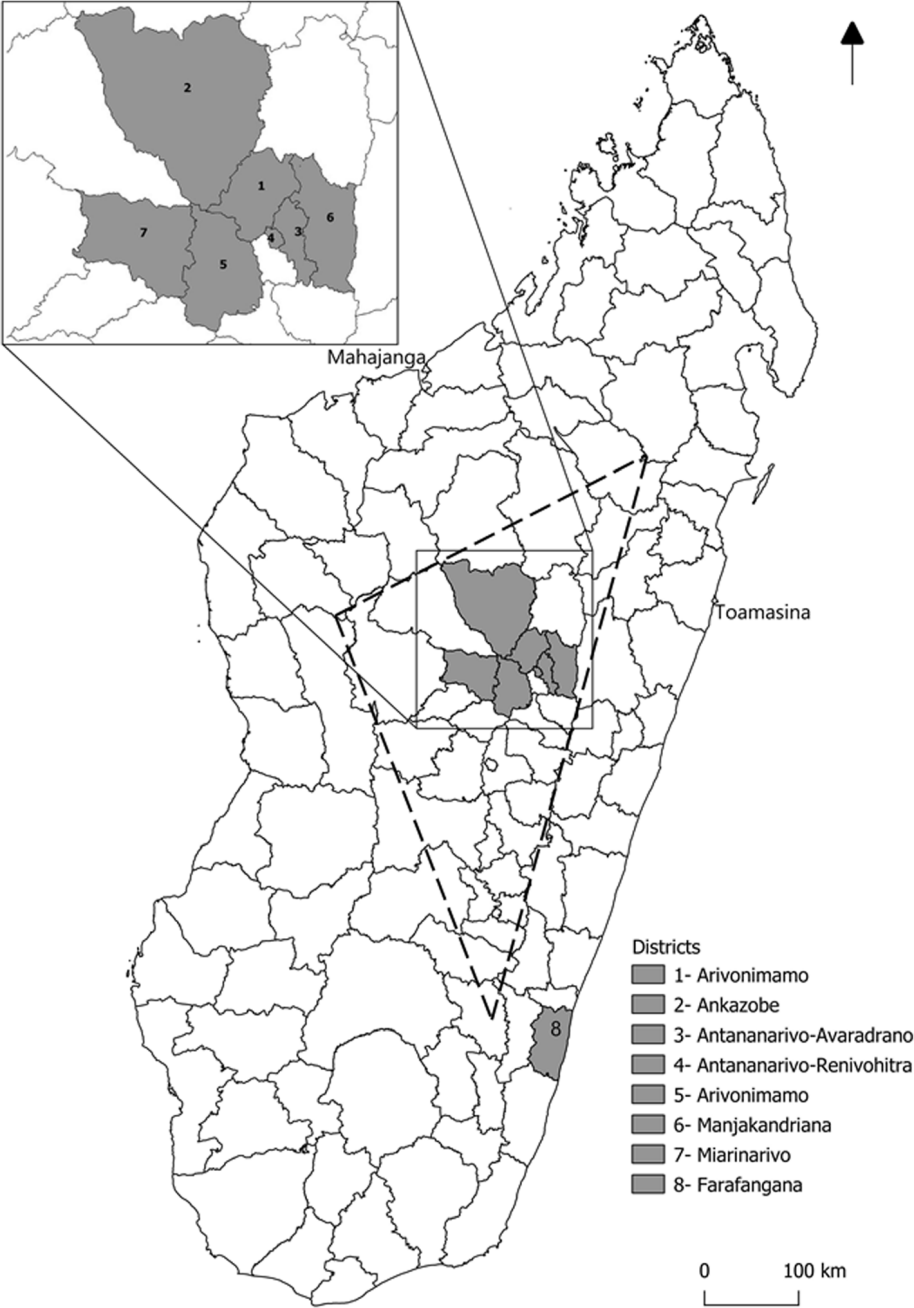
Location of the Districts included in the study sites and plague focus in Madagascar. Dashed line: limits of the main plague-endemic area in the central highlands of Madagascar (altitude > 800 m). This map, created using an open source Geographic Information System QGIS 3.4 software, is freely available for use

We conducted a prospective study between 2005 and 2007 in the district of Ankazobe, Arivonimamo, Manjakandriana and Miarinarivo (Fig. 1) for short-term kinetics of antibodies against *Y. pestis* F1 antigen. Suspected plague patients were enrolled according to their clinical symptoms and the epidemiological contexts.

A retrospective study was carried out between 2006 and 2017 in the district of Ambohidratrimo, Ankazobe, Antananarivo-Avaradrano, Antananarivo-Renivohitra, Arivonimamo, Manjakandriana and Miarinarivo (Fig. 1) for long-term antibodies persistence assessment. Recovered confirmed plague patients were selected from the national plague database of the Central Laboratory for Plague prior to their recruitment to participate to the study.

These districts are located in the main plague focus of the central highlands of Madagascar (Fig. 1).

### Patient recruitment, sample, data collection and serological testing

For the short-term kinetic study, six suspected BP patients were enrolled on their admission day at the primary health center. The clinical diagnosis of plague according to the symptoms and epidemiological context was conducted. As part of the Malagasy Plague National Control Program, patients’ bubo aspirates were tested using antigen F1 detection Rapid Diagnostic Test (F1RDT) [12] and bacteriological culture [13] was performed for confirmation. For each participant, a blood sample was collected at four different time points: on admission, during the first and/ or second week and beyond 1 month after the disease onset. Sera were tested for short-term kinetics of anti-F1 IgG antibody response.

The long-term antibody persistence was assessed on single sera collected from 71 cases selected from the national plague database of the Central Laboratory for Plague and who recovered from plague. These patients were confirmed for plague as *Y. pestis* strain has been isolated from their clinical sample (bubo aspirates or sputum) by bacteriological culture, biochemical identification with API20E gallery strip test (bioMérieux) and bacteriophage lysis test. Time of serum sampling after the documented plague episode was different for each confirmed case. As far as we know, patients we followed-up had not been immunized as plague vaccination was abandoned in the 1950’s in Madagascar, but the possibility that these patients had exposure to *Y. pestis* prior to the study period cannot be ruled out.

For each participant, the following variables were available: age at infection, gender (male/female), district of residence, date of onset, and clinical form (BP/PP).

As negative controls, 10 serum samples were collected from persons with no plague history in an area of endemicity (Antananarivo-Renivohitra District) and 44 serum samples were collected from persons living in non-endemic plague foci (Farafangana District).

All serum samples were tested for the detection of anti-F1 IgG using a laboratory-developed ELISA validated for human plague in Madagascar [9].

Both studies have received the Ethics Committee for Biomedical Research of the Malagasy Ministry of Public Health approvals. Written informed consent was obtained from each participant or legal guardian, including serum sampling.

### Statistical analysis

For statistical analyses, we used the Chi-square test for anti-F1 seroprevalence and association to age class, gender, year of infection or clinical form. Significance was set at *P* < 0.05. The Spearman correlation test was performed to assess the association between anti-F1 IgG titers and the delay from the onset of the disease to the date of sample collection. Student’s *t* test was used for the comparison of delay between seronegative and seropositive patients. Data are presented as median with low and upper quartiles.

## Results

### Short-term kinetics of antibodies against F1 antigen in suspected plague patients

**Fig. 2:**
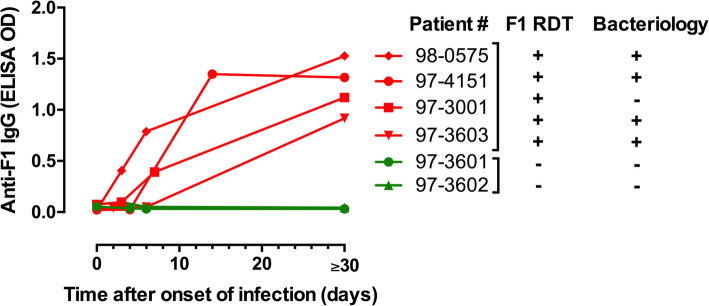
Kinetics of anti- F1 IgG in sera collected from suspected plague patients enrolled in Madagascar, 2005–2007 (*n* = 6). Abbreviations: IgG, immunoglobulin G; ELISA, enzyme-linked immunosorbent assay; OD: Optical density measured at 600 nm (OD threshold = 0.350); F1RDT: Rapid Diagnostic Test for *Y. pestis* F1 antigen detection

Information regarding test results is provided in Fig. 2. Among the 6 suspected plague patients (3 males and 3 females), 3 were confirmed by bacteriology, one was probable (F1RDT positive only) and 2 remained suspected (with symptoms but negative with all performed tests). After the disease onset, anti-F1 IgG titers increased progressively during the first week and reached a maximum at day 30 and beyond. This trend was observed for the 3 confirmed as well as for the only probable patient. No detectable anti-F1 IgG was found for the 2 suspected patients throughout the course of the disease (Fig. 2).

### Persistence of antibodies against F1 antigen in recovered plague patients

**Fig. 3:**
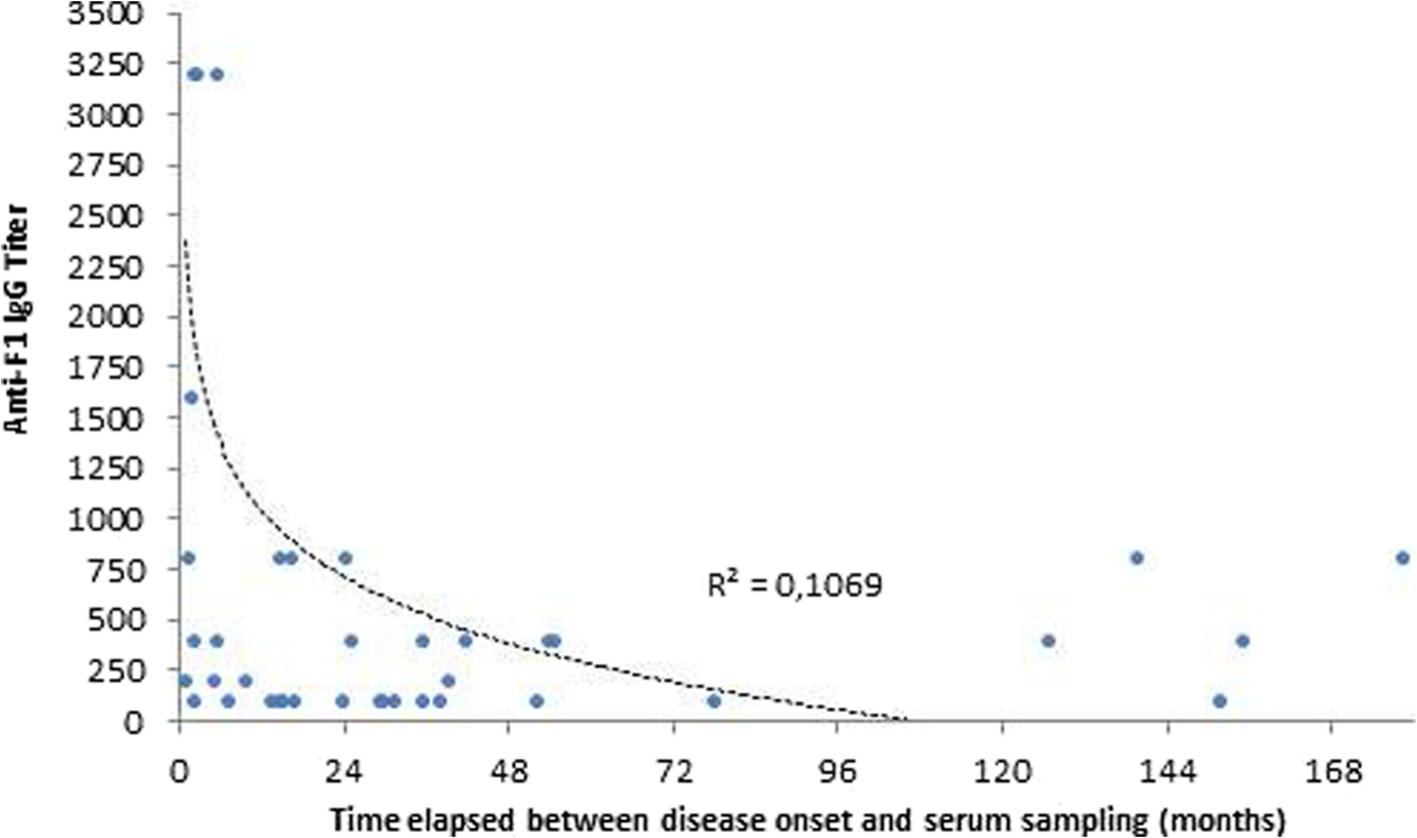
Distribution of anti-F1 IgG titer according to time elapsed between disease onset and serum collection (*n* = 40). The unique titer of 12,800 was not considered when constructing this figure as this is most likely to be an outlier

Among the 71 convalescent plague confirmed patients (37 males and 34 females), 40 (56.3%) still had detectable *Y. pestis* anti-F1 IgG titers in serum samples collected with a median delay of 24 months [IQR 5.57–39.7] after the disease onset, including 16/40 (40%) who were low-antibody responders (87.5% with anti-F1 IgG titer of 100) and 24/40 (60%) who were high-antibody responders (95.6% with anti-F1 IgG titer ranging from 200 to 12,800). The prevalence of anti-F1 IgG was almost the same among patients infected within 5 years (55.7%) and in patients infected from 6 to more than 10 years (60%). For all seropositive patients, there was no correlation between anti-F1 antibodies titer and the time elapsed between the onset of the disease and the time of sampling (Spearman’s rho = − 0.233; *P* = 0.153). Five cases, 1 low-responder and 4 high-responders remained seropositive after a decade with one case after 14.8 years. A negative trend was observed between anti-F1 IgG titer and persistence indicated in months. However, about 10% of variation in anti-F1 IgG titer was only explained by variation in the delay between disease onset and sampling (R^2^ = 0.106; Fig. 3).

**Fig. 4:**
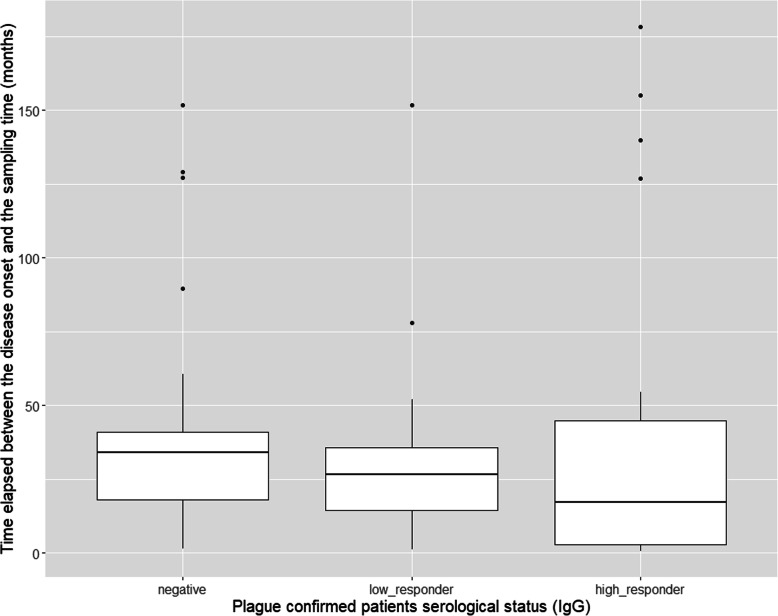
Distribution of plague patients by serological status and time elapsed between disease onset and sampling. High-antibody responders are patients with an OD ≥ 1, and low-antibody responders are patients with an OD < 1. These two groups of patients are seropositive for anti-F1 IgG using ELISA with a threshold value of 0.350

A proportion of confirmed patients were serology negative (31/71) indicating a possible loss of antibody only a few months after disease onset (median 31; IQR 2–152). The mean of time elapsed between disease onset and blood sampling did not differ significantly between seronegative and seropositive convalescent confirmed plague patients (*P* = 0.801). Data regarding plague confirmed cases are summarized in Table 1 and their distribution according to anti-F1 IgG serological status is provided in Fig. 4.

**Table 1:** Recovered plague patients characteristics and serology data (*n* = 71)

Characteristic	Seronegative	Seropositive	***P*** value
**Total patients, n**	31	40	
**PP**	4	1	
**BP**	27	39	
**Median time between disease onset-blood sampling; IQR (months)**	31; IQR 2–152	24; IQR 5.57–39.7	*P* = 0.33
**Median age at onset; IQR**	23; IQR 9–33.5	14; IQR 9.75–23	
**Gender**			*P* = 0.75
**Male**	15	22	
**Female**	16	18	

Abbreviations: *PP* Pneumonic plague, *BP* Bubonic plague, *IQR* Interquartile range

As expected, individuals from endemic areas but without plague history and those from non-endemic areas were all negative for anti-F1 antibodies.

### Epidemiological characteristics of anti-F1 IgG positive cases

To study the relationship between age and the positivity of anti-F1 antibody, recovered patients were divided according to age groups (< 18 and ≥ 18 years-old) when infected. There was no significant difference of seroprevalence for anti-F1 IgG between the two groups (X^2^ = 1.909; *P* = 0.167). Among the 71 convalescent patients, anti-F1 IgG was detected in 18/34 females (52.9%) and 22/37 males (59.4%). The seroprevalence rate is not significantly different between gender (X^2^ = 0.098; *P* = 0.754). According to the clinical form of the disease, BP represents 93% (66/71) versus 7% (5/71) for pneumonic form. Thirty-nine of 66 BP and only one of 5 PP were positive for anti-F1 IgG and no significant difference was observed between seropositivity and clinical forms of the disease (X^2^ = 1.517; *P* = 0.218).

## Discussion

The humoral immune response to *Y. pestis* has been investigated in plague patients either during the acute phase or at the convalescent period of the disease. Very few or no studies on short-term kinetics of antibodies against F1 antigen have been carried out outside of Madagascar [9, 14]. In the present study, the recruitment of suspected patients was often challenging because patients were mostly located in remote areas and data related to the onset of their disease were not always accurate. Therefore, enrollment was lower than expected and only 6 suspected BP patients participated in this short-term study. Overall for confirmed and probable cases, production of anti-F1 IgG appeared during the first week and increased gradually to reach a maximum around day 30 after disease onset. This is in agreement with findings in a previous study performed by Rasoamanana et al [9]. Suspected cases remain negative. During the 2017 PP outbreak, serology was not performed and patients were not followed-up after discharge due to logistical constraints regarding blood collection and sample transport/storage. To address this issue, our study demonstrated that plague confirmation by seroconversion could be obtained from paired sera (at admission and upon discharge) while the patient is still under care at the primary health center, thus preventing the problem of patients’ follow-up. Otherwise, as per the World Health Organization recommendations, plague could be confirmed when a second serum collected at 3 weeks interval achieves a fourfold rise in anti-F1 IgG titer [15].

In Madagascar, long-term antibody persistence was previously studied with paired sera collected from confirmed plague patients during the 1995 plague outbreak in the coastal city of Mahajanga where plague cases were sporadic. Among 11 confirmed patients, only 4 were still seropositive 4 to 6 years post infection [9]. In our study, 71 confirmed patients were recruited from the traditional plague foci located mainly in the central highlands of the island, and only a single serum at the convalescent phase was collected. Assuming that the sera were negative at the onset of the disease as the patients reported not having contracted plague before the episode within this study, more than a half of the recovered patients produced anti-F1 IgG. These antibodies could persist from one to several months after the onset of the disease and anti-F1 IgG titer was high if serum was collected shortly after the disease onset. The higher prevalence in anti-F1 IgG antibodies for patients originating from the central highlands compared to the west coast of Mahajanga may be explained by the fact that plague has been endemic for a long time in the highlands (more than a century) thus the population might have been more exposed to the infection and probably developed a stronger immune response to plague. Indeed, one-third of seronegative confirmed plague patients were from two districts outside the hot spot areas (Antananarivo-Avaradrano and Antananarivo-Renivohitra). However, this IgG persistence time was highly heterogeneous and was not associated with time since infection, suggesting that other factors may be involved.

Plague affects more men than women in Madagascar [16], probably due to higher exposure in agricultural activities. Prevalence of antibodies against *Y. pestis* F1 antigen did not follow this trend as we did not find significant gender-related differences in the persistence of anti-F1 IgG in convalescent plague patients. Age at infection also had no influence on antibody persistence. These observations were also reported in Chinese recovered plague patients [11].

More than 90% of these recovered patients investigated for long-term persistence of antibodies against *Y. pestis* were bubonic cases, which was consistent with previous findings showing that this clinical form was the dominant form of the disease [17]. Out of the 5 patients recovered from PP, only 1 person was seropositive, more than a decade after the disease onset. The limited number of pneumonic convalescent patients did not allow us to assess if there was a difference between persistence of plague antibodies and the clinical form. Moreover, reports of immune response in PP patients are scarce or nonexistent. According to a few papers describing PP outbreak in Madagascar [18–21] and elsewhere [22], most PP patients produced plague antibodies. Pneumonic plague is rare and is often reported from outbreaks with a high mortality rate. Evidently, dead individuals were not blood-sampled post-mortem, serum samples were collected only from surviving patients. In addition in Madagascar, serology is not systematically conducted, unless for retrospective biological confirmation and case definition purpose (confirmation or presumption), days or months after outbreak occurrence, rather than to study antibody persistence. Limitations of our study included the unit to express the anti-F1 IgG results which was different according to the studies performed (Optical Density for short–term versus titer for persistence), the antibody persistence study which was assessed on single serum samples, rather than serial sera and the small size of patients enrolled for the short-term follow-up.

## Conclusions

Our study highlighted that the serological immune response to F1 antigen, which is specific to *Y. pestis*, may be driven by individual responsiveness and could persist for several years and even for more than a decade in both BP and PP recovered patients. In addition, complementary studies including analyses of the cellular immune response to *Y. pestis* are required for the better understanding of long-lasting protection and development of a potential vaccine against plague.

## Data Availability

The datasets used and/or analyzed during the current study are available from the corresponding author on reasonable request.

## Abbreviations

BP: Bubonic plague
PP: Pneumonic plague
F1RDT: F1 Rapid Diagnostic Test
ELISA: Enzyme-linked immunosorbent assay
IQR: Interquartile range
OD: Optical Density
